# The effect of endotoxin adsorber hemoperfusion on microcirculation in patients with severe sepsis and septic shock

**DOI:** 10.1186/2197-425X-3-S1-A810

**Published:** 2015-10-01

**Authors:** Y-C Yeh, Y-C Hsu, A Chao, V-C Wu, W-H Sheng, C-C Kao, Y-J Cheng, NTUH Center of Microcirculation Medical Research (NCMMR)

**Affiliations:** Department of Anesthesiology, National Taiwan University Hospital, Taipei, Taiwan Province of China; Department of Internal Medicine, National Taiwan University Hospital, Taipei, Taiwan Province of China; Department of Internal Medicine, Taipei Medical University Hospital, Taipie, Taiwan Province of China

## Introduction

Microcirculatory dysfunction may result in multiple organ dysfunction during severe sepsis and septic shock.

(1) A meta-analysis of randomized trials showed that polymyxin B hemoperfusion (PMX-HP), plasma exchange, and hemofiltration were associated with lower mortality.

(2) Mesenteric microcirculation was better maintained with polymyxin B hemoperfusion in one rat sepsis study.

(3) However, the effects of polymyxin B hemoperfusion on the microcirculation in patients with severe sepsis and septic shock was unknown.

## Objectives

The aim of this clinical study is to investigate the effect of polymyxin B perfusion on the microcirculation.

## Methods

This multi-center, randomized, controlled study was approved by the National Taiwan University Hospital Research Ethics Committee, which is registered on the ClinicalTrials.gov Protocol registration system (ID: NCT01756755). Forty patients with definitive treatment of severe sepsis and septic shock, which was caused by intra-abdominal infection or proven gram-negative infection, or presence with an Endotoxin Activity Assay (EAA) > 0.6 EAA units, will be randomly assigned to two groups: the Control group and the PMX-HP group. In the Control group, the patients were treated according to the Surviving Sepsis Campaign guidelines. In the PMX-HP group, the patients were treated with an additional treatment with 1 to 2 sessions of polymyxin B hemoperfusion. A sidestream dark-field video microscope was used to record the images of sublingual microcirculation at enrollment (0 h), 24 h and 48 h. The images was analyzed by an investigator blinded to the clinical data and grouping using an automated analysis software (AVA 3.0; Academic Medical Centre, The Netherlands).

## Results

13 patients completed the study. The mean of APCHE II score at enrollment was 18.7 (5.9). 12 patients survived more than 28 days, and 1 patient died in the PMX-HP group. The total small vessel density and perfused small vessel density at 24 h and 48 h were higher in the PMX-HP group than in the Control group (Table 1).

**Table 1:** Table 1

		Control	PMX-HP	P value
n		6	7	
Total SVD (mm/mm2)	0 h	21.7 (2.2)	23.2 (2.4)	0.286
	24 h	19.3 (2.2)	21.8 (0.9)	0.190
	48 h	19.4 (1.5)	22.8 (0.7)	0.001
Perfused SVD (mm/mm2)	0 h	18.6 (4.5)	20.5 (5.5)	0.523
	24 h	18.0 (2.2)	21.2 (1.3)	0.007
	48 h	18.2 (1.6)	22.2 (0.6)	< 0.001

*[Small vessel density (SVD)]*

The differences of microvascular flow index and heterogeneity between the two groups were not significant (Table 2).

**Table 2:** Table 2

		Control	PMX-HP
Microcvascular flow index	0 h	2.6 (0.5)	2.7 (0.4)
	24 h	2.9 (0.2)	3.0 (0.0)
	48 h	2.9 (0.2)	3.0 (0.0)
Heterogeneity index	0 h	0.21 (0.21)	0.21 (0.29)
	24 h	0.17 (0.23)	0.03 (0.08)
	48 h	0.10(0.09)	0.03 (0.08)

*[Microvascular flow and heterogeneity]*

**Figure 1:**
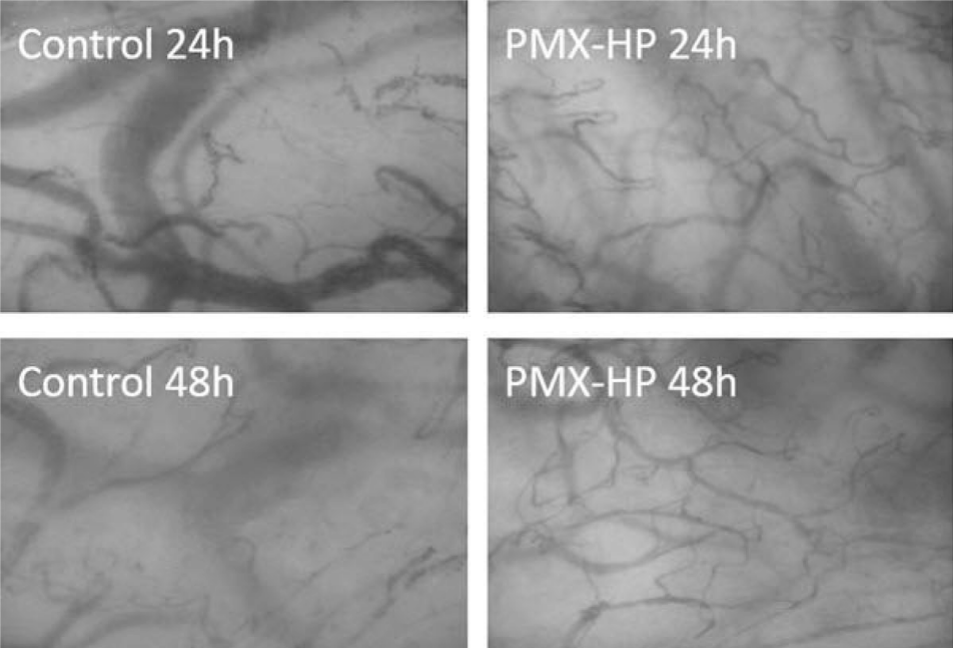
**Sublingual microcirculation**.

The images of sublingual microcirculation at 24 h and 48 h were shown in the Figure 1.

There were no significant difference of mean arterial pressure, dose of vasopressor, serum level of lactate, creatinine, amount of fluid resuscitation, stay in the intensive care unit, and stay in hospital between the two groups.

## Conclusions

In summary, we found that sublingual microcirculation was better maintained with polymyxin B hemoperfusion than conventional treatment in patients with severe sepsis and septic shock.
